# Remote management of osteoporosis in the first wave of the COVID-19 pandemic

**DOI:** 10.1007/s11657-022-01069-x

**Published:** 2022-03-02

**Authors:** Gianmaria Salvio, Claudio Gianfelice, Francesca Firmani, Stefano Lunetti, Rossella Ferroni, Giancarlo Balercia, Gilberta Giacchetti

**Affiliations:** Department of Clinical and Molecular Sciences, Division of Endocrinology, Umberto I Hospital, Polytechnic University of Marche, Via Conca 71, 60126 Ancona, Italy

**Keywords:** Osteoporosis, COVID-19, SARS-CoV-2, Denosumab, Vitamin D, Telemedicine

## Abstract

**Summary:**

We conducted a survey during the first pandemic wave of coronavirus disease 2019 (COVID-19) on a large group of osteoporotic patients to evaluate the general conditions of osteoporotic patients and the impact of the pandemic on the management of osteoporosis, finding high compliance to treatments and low COVID-19 lethality.

**Introduction:**

During the first pandemic wave of coronavirus disease 2019 (COVID-19), 209,254 cases were diagnosed in Italy; fatalities were 26,892 and were overwhelmingly older patients. The high prevalence of osteoporosis in this age group suggests a potential relationship between SARS-CoV-2 infection and bone metabolism.

**Methods:**

In a telephone survey conducted from April to May 2020, patients from the Osteoporosis Center, Clinic of Endocrinology and Metabolic Diseases of Umberto I Hospital (Ancona, Italy), were interviewed to evaluate the general clinical conditions of osteoporotic patients, compliance with osteoporosis medications, COVID-19 prevalence, hospitalization rate, COVID-19 mortality, and lethality.

**Results:**

Among the 892 patients interviewed, 77.9% were taking osteoporosis treatment and 94.6% vitamin D supplementation as prescribed at the last visit. COVID-19-like symptoms were reported by 5.1%, whereas confirmed cases were 1.2%. A total number of 33 patients had been in hospital and the hospitalization rate of those who had not discontinued vitamin D supplementation was less than 4%. There were eight deaths, two with a concomitant COVID-19 diagnosis. The prevalence of severe osteoporosis was 50% in total COVID-19 patients and 87.5% in deceased COVID-19 patients. The overall COVID-19 mortality was 0.2%; lethality was 20%, lower than the national rate of the same age group.

**Conclusions:**

This large group of osteoporotic patients showed high compliance and lower COVID-19 lethality compared to patients of the same age. Novel approaches such as telemedicine can provide critical support for the remote follow-up of patients with chronic diseases also in the setting of routine care.

## Introduction

In late 2019, a novel coronavirus, designated severe acute respiratory syndrome coronavirus 2 (SARS-CoV-2), emerged in Wuhan (Hubei Province, China). It rapidly spread throughout the country and, eventually, the world. The virus often causes bilateral pneumonia (coronavirus disease 2019, COVID-19), which is characterized by a high mortality rate [1]. According to the World Health Organization (WHO) website, by 22 October 2021, laboratory-confirmed cases of COVID-19 in the world had climbed to 241,903,373, and 4,920,570 patients had died [2].

In Italy, the first cases of COVID-19 were confirmed on 30 January 2020. The first outbreak was detected in Codogno (Lombardy) on 21 February 2020, which marks the official start of the first pandemic wave in the country [3]. Italy went into lockdown until 4 May 2020, which marked the official end of phase 1 of the pandemic. By this date, 209,254 cases had been diagnosed in the country and 26,892 patients had died [4]. A summer lull was followed by a new wave of infections. Indeed, in November 2020, more than 20,000 new cases were confirmed each day, making a new lockdown increasingly probable. Elderly people, who according to early reports were at higher risk of dying from SARS-CoV-2 worldwide, were invited to leave their homes only if absolutely necessary [5]. Also in Italy, more than 80% of the 35,000 fatalities involved individuals aged 70 years or older [2].

Osteoporosis is a frequent disease among the elderly. It is a systemic disorder characterized by a progressive quantitative and qualitative alteration of the bone mass that involves an increased fracture risk even without trauma and whose incidence increases with age [6, 7]. In Italy, where more than 460,000 new fragility fractures are treated each year [8], osteoporosis affects about 3.5 million women and 1 million men and is the third commonest chronic disorder after hypertension and arthritis/osteoarthritis [9].

Our group has recently published a review on bone metabolism in SARS-COV-2 patients [10]. However, clinical data on the fracture risk associated with COVID-19 are still limited; the role of vitamin D supplementation, which is commonly prescribed to osteoporosis patients, is debated; and information on the effects of other drugs used to treat osteoporosis is scanty.

According to a recent report, fracture patients may be at increased risk of contracting COVID-19 [11], and in case of infection, they experience a worse outcome, both in terms of length of hospitalization and mortality [12]. On the other hand, it has been reported that home confinement involved a reduced overall incidence of fractures during the first wave of the pandemic compared with previous years [13, 14]. Indeed, a very recent meta-analysis has shown a 43% (range 35–50%) reduction in the overall incidence of fractures from December 2019 to May 2020 that was primarily due to a reduction in sports-related fractures, although a relative increase in work-related fractures, high-energy falls, and domestic accidents was also observed. Furthermore, despite a high similarity of fracture sites between the pandemic and the pre-pandemic periods, hand fractures declined during the pandemic, whereas there was a borderline significant increase in femoral fractures. The major finding emerging from this study was that 30-day mortality associated with fractures increased significantly during the pandemic period (9% vs 4%, OR 1.86 [1.05, 3.27], *p* = 0.03) [15].

Taken together, these data indicate that fracture prevention is a key goal in osteoporosis treatment and that, during the pandemic, additional effort is required to ensure continuity of care and maximize patient compliance with treatment. To provide indications for the optimal management of endocrine conditions during the pandemic, the European Society of Endocrinology has published specific guidelines for diabetes [16], thyroid diseases [17, 18], pituitary tumors [19], electrolyte disturbances [20], and bone metabolism disorders [21]. The latter place a strong emphasis on “remote follow-up” such as telephone contacts and video consultations. We report the experience of a regional reference center for the diagnosis and treatment of bone metabolism disorders in managing osteoporotic patients during the first wave of the pandemic.

## Methods

In a telephone survey conducted from 1 April to 4 May 2020, we contacted all the patients (or, in case of non-availability, their family members) who should have been visited at the Osteoporosis Center of the Clinic of Endocrinology and Metabolic Diseases of Umberto I Hospital (Ancona, Italy) from March to June 2020. During this period, non-urgent visits were suspended due to the national lockdown and clinical checks were postponed until a later date. Thus, the survey was an opportunity to ensure continuity of care for these patients. On the same occasion, in fact, useful information was provided to overcome the limitations imposed by the lockdown (e.g., access to drugs and laboratory analysis and management of any side effects of therapies). The interview (conducted by F.F., C.G., S.L., and R.F.) evaluated primarily their health status and the incidence of COVID-19 cases. After collecting their verbal informed consent to participate and to examine their medical chart, we asked for the following information: age, Area Vasta (AV; i.e. local health service) of origin (AV1 to AV5), current osteoporosis treatment, vitamin D supplementation (prescribed at the usual dosage for the treatment of osteoporosis), previous fragility fractures (femoral, vertebral, other), cardiovascular comorbidities (diabetes mellitus, hypertension, a history of heart disease), and secondary causes of osteoporosis (renal transplant or adjuvant hormone therapy for breast or prostate cancer). Severe osteoporosis was reported according to the definition of bone density value below the − 2.5 SDS of T-score and the presence of one or more fragility fractures. Patients were also asked about symptoms suggestive of COVID-19 infection (e.g., fever > 37.5 °C), diagnosis of SARS-CoV-2 infection confirmed by a nasopharyngeal swab with molecular analysis, and hospitalization for any cause in the first phase of the pandemic (21 February–4 May 2020). Any deaths reported by family members were recorded. The serum 25-OH-vitamin D levels of COVID-19 patients were recorded if available.

### Statistical analysis

Statistical analysis was performed with SPSS 23.0 software (SPSS Inc., Chicago, IL, USA). The primary objective of the study was to report descriptive statistics of the information collected. The secondary objective was to study the association between these and the outcomes of COVID-19. Data were collected by telephone and by consulting clinical records where available. Continuous variables were subjected to Shapiro–Wilk normality tests if their mode of distribution was not evident from the graphical representation by histogram and/or normality plot. If normal, distributions were described in terms of mean and standard deviation; otherwise, median and interquartile range were used. Categorical variables were presented in terms of frequency and percentage. Bivariate correlations were investigated by Spearman’s test. Logistic regression was used to investigate the effect of demographic characteristics (age as continuous and AV of origin as categorical variables) and vitamin D supplementation or comorbidities (as categorical variables) on risk of hospitalization, COVID-19, and death. Statistical significance was set at *p* < 0.05.

## Results

**Fig. 1:**
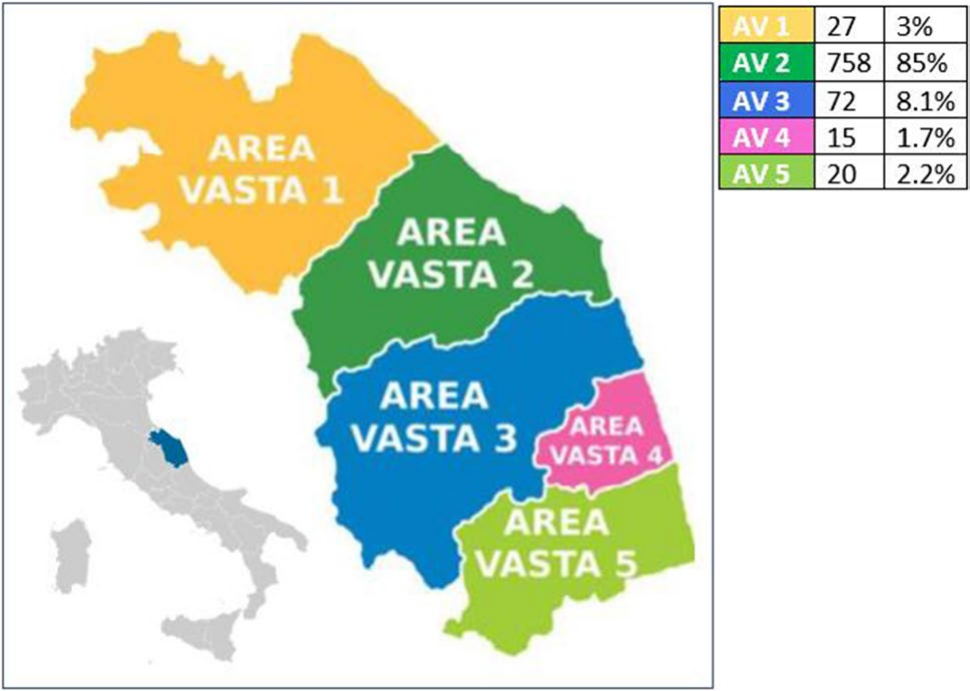
Local health service of MarcheRegion and distribution of patients. AV= Area Vasta

Of a total of 910 interview subjects, 892 provided consent to participate in the survey (response rate 98%), including 785 women (88%) and 107 men (12%) whose mean age was 70.9 ± 11.4 years. Most belonged to AV2 (758, 85%), followed by AV3 (72, 8.1%), AV1 (27, 3%), AV5 (20, 2.2%), and AV4 (15, 1.7%) (Fig. 1). All data are summarized in Table 1.

**Table 1:** Population characteristics

	Whole sample (n=892)	Fever (n=44)	COVID-19 (n=10)	Deceased (n=8)
Gender				
F	785 (88%)	40 (90.9%)	10 (100%)	7 (87.5%)
M	107 (12%)	4 (9.1%)	0 (0%)	1 (12.5%)
Age	70.9 ± 11.4	69.1 ± 11.7	79.9 ± 8.1	84.7 ± 3.4
Origin				
AV1	27 (3%)	0 (0%)	0 (0%)	0 (0%)
AV2	758 (85%)	40 (90.9%)	9 (90%)	8 (100%)
AV3	72 (8.1%)	2 (4.5%)	1 (10%)	0 (0%)
AV4	15 (1.7%)	2 (4.5%)	0 (0%)	0 (0%)
AV5	20 (2.2%)	0 (0%)	0 (0%)	0 (0%)
Fever	44 (5.1%)	-	9 (90%)	2 (25%)
Treatment (except vit. D)				
None	195 (22.1%)	10 (22.7%)	0 (0%)	0 (0%)
Denosumab	502 (56.8%)	28 (63.7%)	8 (80%)	8 (100%)
Teriparatide	39 (4.4%)	0 (0%)	0 (0%)	0 (0%)
Oral bisphosphonates	102 (11.6%)	3 (6.8%)	0 (0%)	0 (0%)
Intravenous bisphosphonates	45 (5.1%)	3 (6.8%)	2 (20%)	0 (0%)
Vitamin D	833 (94.6%)	39 (88.6%)	9 (90%)	8 (100%)
Diabetes mellitus	80 (9.4%)	6 (13.6%)	2 (20%)	1 (14.3%)
Hypertension	454 (52.8%)	25 (56.8%)	4 (40%)	5 (71.4%)
Heart disease	140 (16.3%)	6 (13.6%)	2 (20%)	3 (42.9%)
Kidney transplant	71 (8%)	3 (6.8%)	0 (0%)	0 (0%)
Hormone therapy for cancer	336 (38.4%)	18 (40.9%)	3 (30%)	0 (0%)
COVID-19 infection	10 (1.2%)	9 (20.9%)	-	2 (33.3%)
Fractures (any)	396 (44.8%)	15 (34.1%)	4 (40%)	7 (87.5%)
Fractures (femoral)	44 (5%)	4 (9.1%)	1 (10%)	0 (0%)
Fractures (vertebral)	364 (41.3%)	16 (36.4%)	3 (30%)	7 (87.5%)
Fractures (other)	99 (14.3%)	11 (34.4%)	5 (83.3%)	3 (50%)
Deceased	8 (0.9%)	2 (4.5%)	2 (20%)	-
Hospitalization	33 (3.9%)	11 (25%)	7 (70%)	3 (50%)
Severe osteoporosis	408 (45.7%)	17 (38.6%)	5 (50%)	7 (87.5%)

**Fig. 2:**
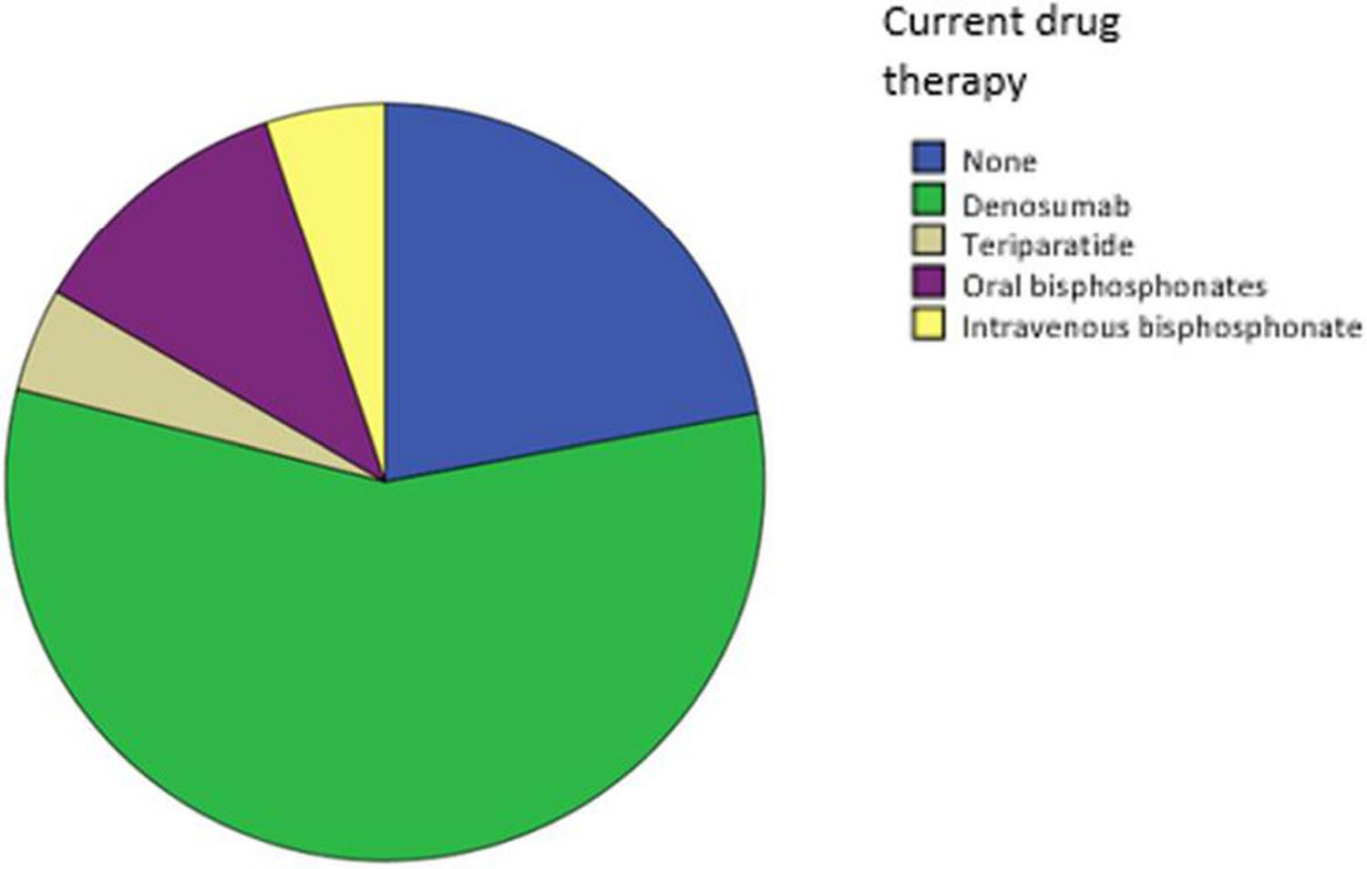
Study population according to current drug treatment

Information on current pharmacological treatment was provided by 882 patients. Excluding vitamin D supplements, 195 subjects (22.1%) were not on a specific osteoporosis drug, whereas 687 subjects (77.9%) had an active prescription and were regularly taking their medication. Most participants (501, 56.8%) were on denosumab, followed by oral bisphosphonates (102, 11.6%), intravenous bisphosphonates (42, 5.1%), and teriparatide (39, 4.4%) (Fig. 2). Notably, 94.9% were also taking vitamin D supplements.

The most frequent comorbidity was hypertension (454, 52.8%), followed by a history of breast or prostate cancer that was being treated with adjuvant hormone treatment (336, 38.4%); a history of heart disease (140, 16.3%); diabetes mellitus (80, 9.4%); and kidney transplant (71, 8%). Of the 892 participants, 42% had at least one comorbidity, 25% had 2 comorbidities, and 9% had 3 or more comorbidities. As regards fractures, 396 patients (44.8%) had had at least one previous fracture; altogether they had suffered 44 femoral fractures, 364 vertebral fractures, and 99 fractures at another site. Based on their clinical history, 408 patients (45.7%) were classified as having severe osteoporosis.

Before the interview, 44 subjects (5.1%) had had a febrile episode, 10 (1.2%) had been diagnosed with COVID-19, and 33 (3.9%) had been in hospital (any cause). Eight (0.9%) of the patients sought for the interview had died. Interestingly, we have observed few cases of COVID-19 in the face of a high prevalence of denosumab use and, consequently, a low incidence of COVID-19 in patients treated with denosumab (8/502, 1.6%).

The COVID-19 patients (all female) were significantly older than non-COVID-19 subjects (79.9 ± 8.1 vs 70.8 ± 11.4 years, *p* = 0.01) but showed no significant differences in terms of comorbidities. Fever was a frequent presenting symptom of infection and was reported in 9 of the 10 patients. The overall COVID-19 mortality was 0.2%; lethality was 20%. The complete data of the COVID-19 patients are presented in Table 2.

**Table 2:** Multivariate analysis

	Dependent variables						
		Hospitalization	P	COVID-19	p	Death	p
Model #1	Age	1.027 (0.994-1.062)	0.11	1.106 (1.022-1.197)	0.01	1.228 (1.078-1.400)	0.002
	Gender	1.015 (0.346-2.997)	0.98	-	0.99	1.423 (0.163-12.399)	0.75
	Vitamin D supplementation	0.271 (0.098-0.750)	0.01	0.363 (0.043-3.040)	0.35	0.706 (0.506-1.256)	0.85
Model #2	Diabetes mellitus	1.418 (0.504-3.986)	0.51	2.830 (0.572-13.990)	0.20	1.570 (0.170-14.525)	0.69
	Hypertension	0.899 (0.415-1.945)	0.79	1.383 (0.369-5.181)	0.33	1.410 (0.250-7.966)	0.69
	Heart disease	3.843 (1.797-8.220)	0.001	1.116 (0.224-5.563)	0.89	2.600 (0.517-13.064)	0.25
	Kidney transplant	1.482 (0.444-4.946)	0.52	-	0.99	-	0.99
	Hormone therapy	1.129 (0.511-2.492)	0.76	0.546 (0.130-2.294)	0.41	-	0.99
	Fractures	0.730 (0.330-1.617)	0.44	0.626 (0.162-2.424)	0.49	-	0.99

Of the 8 patients who died (7 women, 1 man), 2 had COVID-19 and had been admitted to hospital. Of the remaining 6 deceased patients, one had been admitted to hospital and 5 died at home. The mean age of these 8 patients was significantly higher than that of the 884 patients who survived (84.7 ± 3.4 vs 70.8 ± 11.2 years, *p* < 0.001). Moreover, 7 out of 8 had a history of vertebral fractures. The frequency of severe osteoporosis was significantly higher among the deceased patients (87.5% vs 45.4%, *p* = 0.03).

The 2 patients who died of COVID-19 infection were both female and came from AV2. Both developed fever and were taking denosumab and vitamin D supplements; their family members said they were taking vitamin D, although dosing just before the lock down indicated vitamin D deficiency. One was 89 years old and suffered from hypertension, heart disease, and previous fractures (of which at least one was a vertebral fracture), which led her to be classified as having severe osteoporosis. The other patient was 86 years old and had a history of multiple fractures (at least one vertebral fracture) without other comorbidities. Both patients died in hospital.

According to the logistic regression model considering only vitamin D supplementation, the supplement had a protective effect against the risk of hospitalization (OR 0.31, CI 0.11–0.84, *p* = 0.02) but no effect against the risk of infection or death.

The multivariate analysis was performed with different models with “hospitalization,” “COVID-19,” and “death” as the outcome variables (Table 3).

**Table 3:** Data of the COVID-19 patients

Patient	Age (years)	Origin	Fever	Therapy	Vitamin D supplementation	Comorbidities	Fractures	Severe osteoporosis	Hospitalization	Death	Serum 25-OH- Vitamin D (ng/ml)
#1	87	AV2	Yes	Denosumab	Yes	HT	No	Yes	No	No	25
#2	79	AV2	Yes	Denosumab	Yes	-	V, F	Yes	No	No	22
#3	64	AV2	Yes	IVBP	Yes	HT	No	No	No	No	29
#4	81	AV2	No	Denosumab	Yes	-	No	No	Yes	No	19
#5	80	AV2	Yes	Denosumab	No	DMHTHeart diseaseHTC	No	No	Yes	No	18
#6	70	AV2	Yes	Denosumab	Yes	HT	No	No	Yes	No	20
#7	76	AV2	Yes	Denosumab	Yes	HTC	V, O	No	Yes	No	26
#8	87	AV3	Yes	IVBP	Yes	HTHTC	V, O	Yes	Yes	No	18
#9	89	AV2	Yes	Denosumab	Yes	HTHeart disease	V, O	Yes	Yes	Yes	12
#10	86	AV2	Yes	Denosumab	Yes	-	V, O	Yes	Yes	Yes	18

IVBP: intravenous bisphosphonates; V: vertebral; F: femoral; O: Other sites; HT: hypertension; DM: diabetes mellitus; HTC: hormone therapy for cancer

According to model #1, which considered age, gender, and vitamin D supplementation, the supplement was confirmed to have a protective role against the risk of hospitalization (OR 0.271, CI 0.098–0.750, p = 0.01) but not against the risk of infection or death, whereas age was a risk factor for both COVID-19 infection and death (OR 1.106, CI 1.022–1.197, *p* = 0.01 and OR 1.228, CI 1.078–1.400, *p* = 0.002, respectively).

Model #2 considered the following comorbidities: diabetes, hypertension, heart disease, kidney transplant, cancer, and fractures. It confirmed the strong association between heart disease and risk of hospitalization (OR 3.8 (1.8–8.2), *p* = 0.001).

## Discussion

By the end of our survey, on 4 May 2020, 209,254 cases of COVID-19 had been verified in Italy. Within this retrospective survey, patients had a median age of 62 years (> 70 years in 39.1% of cases); females accounted for 53.1%, and lethality was 12.9%. However, lethality in the older age groups, i.e., patients aged 70–79, 80–89, and > 90 years, was respectively 24.5%, 29.5%, and 25.5% [4]. In the Marche region, infections had been 6,363 (2,571 in AV1, 1,819 in AV2, 1,034 in AV3, 451 in AV4, and 284 in AV5) with 932 deaths and a lethality rate of 14.7%, which was slightly higher than the national mean [22]. By 4 May, 10 of our 892 interviewees had contracted COVID-19 infection and two had died from it. The lethality rate was 20%, which was considerably less than the national mean for the same age group.

Since our hospital is an integrated Regional Reference Center in the osteoporosis prevention and treatment network, a high percentage of patients (38.4%) had a history of hormone-sensitive cancer and were on adjuvant hormone treatment (aromatase inhibitors and LH-RH analogs for breast cancer and LH-RH analogs and 5α-reductase inhibitors for prostate cancer). This explains the large number of subjects who were on antiresorptive therapy with denosumab (56.8%), which according to the Italian Medicines Agency is the first-line treatment for these conditions. Denosumab is a fully human monoclonal antibody that binds to and inhibits the ligand of RANK (RANKL) — a surface protein produced primarily by osteoblasts, periosteal cells, and preosteoclasts — which stimulates osteoclast formation and action by binding to nuclear factor kappa-B activator receptor (RANK), eventually resulting in bone resorption [23]. The RANK-RANKL system has extensively been studied in relation to osteoporosis caused by chronic inflammatory diseases such as rheumatoid arthritis and ankylosing spondylitis, which are frequently associated with local and systemic loss of bone mineralization and entail an increased fracture risk [24]. In fact, RANK-RANKL is an important immune system hub: originally identified as a mediator of T-lymphocyte activation [25], it is an important target of several proinflammatory cytokines like interleukin-1, interleukin-18, and TNF-α and induces immune-mediated upregulation of osteoclastic activity, which in turn promotes bone catabolism [26]. Since SARS-CoV-2 triggers a massive release of proinflammatory molecules by the immune system (the so-called cytokine storm) [27], increased bone resorption mediated by RANK-RANKL system activation can be expected in COVID-19 patients, and combined with the effects of steroid therapy and prolonged immobilization [10] has the potential to raise the incidence of fragility fractures in patients who have recovered from the infection. In this context, RANK-RANKL system inhibition by denosumab may play a protective role against inflammation-related bone resorption. Notably, initial concerns of a putative generic increase in infection susceptibility, related to immunomodulation inhibition in extraskeletal tissue by RANK-RANKL, have been ruled out by clinical trials [23]. Although there are currently no purpose-built studies to evaluate the safety and efficacy of denosumab during the pandemic, current European guidelines recommend current therapy continuation [21]. In a telephone survey of 42 patients by Formenti et al., none of the 26 subjects receiving denosumab had been diagnosed with COVID-19, although one developed symptoms of respiratory tract infection that resolved in 3 days (no nasopharyngeal swab) [28]. In a recent work involving more than 2000 patients treated for rheumatic diseases, 264 (12.6%) received denosumab; of these, only 8 (7.34%) contracted COVID-19 out of a total of 109. Analysis of their medications (which included antiresorptives, antidepressants, antiepileptics, and calcium and vitamin D supplements) demonstrated that denosumab and intravenous zoledronic acid were associated with a 40% lower risk of COVID-19 infection (RR = 0.58; CI95% 0.28, 1.22 and RR = 0.62; CI95% 0.27, 1.41, respectively) [29]. Our data are largely in line with this study, except that we found an even lower incidence of COVID-19 cases (1.6%) in patients receiving denosumab. This confirms its clinical safety during the pandemic and actually suggests a protective role for it against the infection, even though prospective studies are clearly needed to gain insight into this important aspect. This result could be considered apparently in contradiction since denosumab was the most used drug in our population (56% of the total); it was more likely that the few deaths detected fell into that category. Furthermore, from a statistical point of view, the randomness of this observation cannot be captured. The other treatment groups, in fact were too different in numerosity to make comparisons, and we cannot rule out that what we observed was merely causal.

In our patient sample, age was a negative prognostic factor for both the risk of COVID-19 infection (1.106, CI 1.022–1.197, *p* = 0.01) and the risk of death (OR 1.228, CI 1.078–1.400, *p* = 0.002), as also suggested by the mean age of the infected (79.9 ± 8.1) and deceased (84.7 ± 3.4) subjects. This is in line with the most recent WHO reports. In contrast, none of the comorbidities tested in our analysis played a significant role in these two outcome measures. Notably, a history of heart disease was found to involve an almost fourfold risk of hospitalization (OR 3.8 (1.8–8.2); *p* = 0.001). However, the absence of a relationship between this parameter and death or COVID-19 infection does not exclude that the increased hospitalization rate resulted from clinical situations unrelated to COVID-19 or from an increased focus on subjects considered at higher risk during the pandemic.

A very interesting finding was that vitamin D supplementation protected patients against the risk of hospitalization (0.271, CI 0.098–0.750, *p* = 0.01). Nearly 95% of the participants in our study were taking vitamin D supplements at the time of the interview. Previous observational studies have highlighted an increased susceptibility to respiratory infections in subjects with vitamin D deficiency [30]. In addition, vitamin D deficiency has been hypothesized to contribute directly to the development of acute respiratory distress syndrome (ARDS) [31] and autoimmune endocrine disorders [32], and a recent meta-analysis suggested that the administration of vitamin D may improve lung function in patients with asthma [33]. These findings may be related to the immune modulating role of vitamin D, since in vitro studies have demonstrated that calcitriol stimulates monocyte to macrophage differentiation, reduces the production of proinflammatory factors by activated macrophages, and upregulates the expression of several antimicrobial peptides (e.g., CAMP and β-defensins) by innate immune cells, thus enhancing the initial response to infection and protecting the organism from excessive cytokine activation [34, 35]. Low vitamin D levels have been reported to be an independent risk factor for hospitalization and COVID-19 [36] and to be associated with increased susceptibility to SARS-CoV-2 infection [37–39] as well as worse COVID-19 outcomes, such as a greater need for noninvasive ventilation and high dependency units [40] and greater pulmonary involvement and mortality [41]. However, several factors like age, male gender, socioeconomic level, obesity, diabetes, and hypertension are also associated with hypovitaminosis D and their possible confounding effect should be considered [42]. In addition, it is unclear whether vitamin D supplementation can reverse this trend [43], although a recent exploratory study has reported promising results [44]. Therefore, while the indication for supplementation in case of hypovitaminosis D and osteoporosis remains valid, also during the pandemic [21], prospective randomized controlled trials would be useful to define the role of hypovitaminosis D correction in patients with SARS-CoV-2 infection.

The main strength of our study is sample numerosity. With almost 900 interviewees, ours is one of the most representative real-life experience studies in the literature. In addition, direct contact with the physician, who provided reassurance and recommended patients to take their medications, was much appreciated and favorably affected compliance. In this way, despite the closure of the outpatient clinic, patients were able to keep in contact with their reference specialist. For these reasons, in anticipation of the next pandemic waves, we are strengthening the telemedicine system, which is expected to prove useful also in routine care and follow-up of our patients.

We would also like to emphasize that the present study was not designed as an epidemiological survey but as a report on our clinical activity during the COVID-19 pandemic. Since nasopharyngeal swabs were scarce during the first pandemic wave, the number of patients with a confirmed diagnosis of COVID-19 was certainly lower than the number of real patients. We partially tried to compensate for this bias by including patients with a possible diagnosis of COVID-19, who had developed mild symptoms, in the category of patients with COVID-like symptoms. The patients who died at home, on the other hand, had performed a swab and were certainly negative.

The main study limitation is its cross-sectional design, which does not allow establishing a cause-effect relationship of treatment compliance, vitamin D supplementation, and susceptibility to COVID-19 infection and/or effects of COVID-19 infection on bone metabolism and fracture risk. Prospective studies of large populations are required to provide the needed additional evidence.

## Conclusions

The present study, conducted on a large group of remotely monitored subjects, provided useful information on the experience of a regional referral center for the diagnosis and treatment of osteoporosis in managing bone metabolism disorders during the first wave of the COVID-19 pandemic.

Our frail patients followed up by phone felt reassured, they showed high treatment compliance, and experienced a lower COVID-19 lethality rate than patients of the same age; those who had not discontinued their vitamin D supplement also had a reduced hospitalization rate. The results of our survey, especially the low incidence of cases of SARS-CoV-2 infection, support a possible protective role of vitamin D and denosumab against severity of COVID-19, although they need to be verified by large prospective studies.

Last but not least, the study highlighted the critical value of telemedicine in the context of the pandemic as well as in the routine monitoring and care of old and frail patients and of those with chronic disease.

### Ethics approval

The studies conducted in this article do not involve human participants or animals.
